# Accelerated therapeutic development during COVID-19: insights, regulatory strategies, and recommendations for future pandemic preparedness

**DOI:** 10.3389/fmed.2025.1482035

**Published:** 2025-04-15

**Authors:** Miyuki Katayama, Mayumi Shikano

**Affiliations:** ^1^Graduate School of Pharmaceutical Sciences, Tokyo University of Science, Tokyo, Japan; ^2^Faculty of Pharmaceutical Sciences, Tokyo University of Science, Tokyo, Japan

**Keywords:** COVID-19, SARS-CoV-2, emergency use authorization, pandemic, clinical development, 100 days mission, streamlined development

## Abstract

**Background:**

The clinical development of therapeutics for COVID-19 proceeded at an extraordinary pace. Given the lack of studies evaluating this experience systematically, we analyzed the clinical development methods for COVID-19 therapeutics to determine strategies for shortening the clinical development period in preparation for future pandemics.

**Methods:**

We confirmed the US-FDA review documents for fourteen products that underwent Emergency Use Authorization (EUA) in the US during the COVID-19 pandemic to examine the time required for clinical development and regulatory review and the submitted data for EUA.

**Results:**

Six of the fourteen products with clinical study data for other indications were evaluated in fewer studies than new molecular entities. The application data for each product included the stipulated content, and placebo-controlled comparative studies were included for all products. Clinical development measures were adopted, including adaptive protocol design, nonsequential phase development, and clinical dose adaptation based on non-clinical study results.

**Conclusion:**

Products with clinical study data for other indications are advantageous for early approval. However, early approval of new molecular entities is also important because they may not be sufficiently effective against new infectious diseases. It would be effective to approve a product promptly for a limited target population at first and then gradually expand it as data becomes more abundant. To prepare for future pandemics, we recommend establishing a framework for identifying candidates from existing products, managing and disseminating information in emergencies at various levels, and clarifying the conditions for applying regulatory flexibility to encourage pharmaceutical companies to make early decisions regarding clinical development.

## 1 Introduction

As the clinical development of therapeutics for coronavirus disease 2019 (COVID-19) was extremely urgent, the first product became available in Europe, the US, and Japan only 3 months after the World Health Organization’s (WHO) Public Health Emergency of International Concern (PHEIC) declaration was issued (1, 2). This is astonishing, considering that the normal process of new drug research and development takes approximately 9 years from the start of the clinical study to regulatory approval (3).

During the clinical development of therapeutics for COVID-19, efforts have been made to make these products readily available. One such initiative was the US Emergency Use Authorization (EUA) System. Under Section 564 of the Federal Food, Drug, and Cosmetic Act, the US Food and Drug Administration (FDA) may approve the use of unapproved drugs or expand the indications for approved drugs in emergencies when benefits outweigh the risks and efficacy can be estimated based on limited data, even before the completion of clinical studies (4, 5). Similarly, the European Union (EU) has a system called Compassionate Use, Conditional Marketing Authorization, and in Japan, the Special Approval System (2). During the pandemic, these programs allowed early access to several medications in each region.

The 100 Days Mission was presented as an international goal to achieve the practical use of medical countermeasures, including the approval of rapid diagnostics, safe and effective vaccines, and establishing treatment methods within 100 days after the WHO declared a PHEIC (6). It was proposed at the G7 summit held in the UK in June 2021 in preparation for the next pandemic based on the issues revealed by the COVID-19 pandemic. The period set at 100 days was based on the idea that if diagnostics, vaccines, and therapies could be put into practical use and supplied to the world in a shorter period, the number of infected people and deaths could be reduced worldwide, and a future epidemic could be brought under control faster. To prepare for the next pandemic, it is important to apply what we have learned from developing therapeutics for the COVID-19 pandemic; however, no study has evaluated this aspect systematically.

This study reviews challenging clinical development cases during the COVID-19 pandemic as a social experiment and proposes strategies for preparing for future pandemics. We focused on the clinical development of therapeutics for COVID-19 by investigating the development timelines and materials for regulatory review, including clinical data packages of therapeutics for COVID-19 that became available during the pandemic period in the US, to clarify the characteristics of the clinical development of these products and draft proposals for efficient development for future pandemics. We chose the US rather than the EU or Japan because the EU has multiple processes that allow for the emergency use of medical products, including Compassionate Use Opinion and Conditional Marketing Authorization, which makes it challenging to deal with the data uniformly. There is a special approval process for emergencies in Japan; however, the approval conditions include marketing authorization in a foreign country or region, and we thought that approval would be affected by the timing of foreign authorization.

## 2 Materials and methods

We retrieved the submission and approval dates of therapeutics from regulatory review documents, identified the clinical studies used for regulatory evaluation, verified the study registration dates, and assessed the duration of each clinical development process. Additionally, we examined the data included in the application data package. Since COVID-19 is a novel disease with a clearly defined date of emergence, unlike many other diseases, we were able to determine both the study start-up period and the overall duration of the clinical development process.

This was a retrospective cross-sectional study, and the data collection and analysis were performed from October 2023 to August 2024. As this study did not include data or information derived from human participants, it was not reviewed by the Institutional Review Board of the authors’ university.

### 2.1 Identification of therapeutic products for COVID-19

We focus on pharmaceuticals for COVID-19 authorized through the EUA process in the US, from the WHO Declaration of the COVID-19 PHEIC (January 30, 2020) to the Declaration of Termination (May 5, 2023), which were identified on the FDA website (1, 7, 8). The pharmaceutical products were identified and classified based on their characteristics, including their non-proprietary names, brand names, administration route, treatment target, and EUA submission and authorization dates. Additionally, for each therapeutic, we confirmed the study registration dates from trial registries, phases, documented study phase and designs, and examined data included in the application data package, as detailed in the following sections.

### 2.2 Time required for clinical development and regulatory review of pharmaceuticals

The dates of EUA application and authorization by the FDA for the identified drugs and biological therapeutic products were confirmed using the Center for Drug Evaluation and Research (CDER) review documents (9–23). Clinical study data for COVID-19 therapeutics submitted for regulatory review were also identified based on review documents, and the study registration date was obtained from clinical study registration sites, ClinicalTrials.gov, and the EU Clinical Trials Database (24, 25). The period from the PHEIC declaration to the date of EUA issuance for each product and the period of each process constituting it were also calculated.

Each process was defined as follows: (A) study start-up period, from the date of the PHEIC declaration to the earliest registration date of the submitted clinical studies; (B) clinical study period, from the earliest registration date of the submitted studies to the date of EUA submission; (C) review period, from the date of EUA application to the date the EUA was issued; and (D) duration of clinical development, as the sum of (A), (B), and (C), from the date of the PHEIC declaration to the date of the first EUA issued for COVID-19 treatment. This analysis included only studies registered at clinical study registration sites after the PHEIC declaration and excluded those that started before the declaration for other indications.

**Figure 1:**
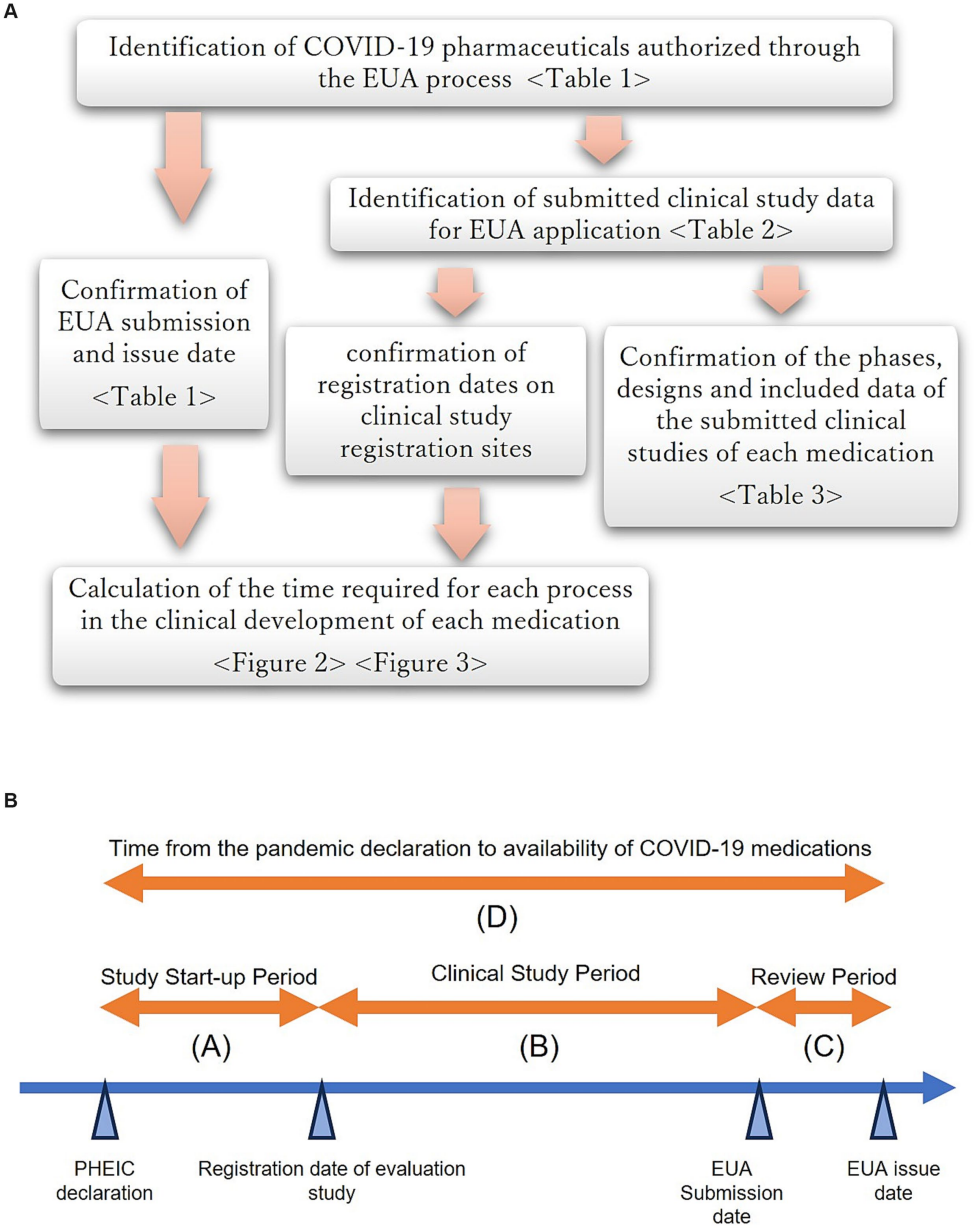
**(A)** Overview of the study flow. **(B)** Definition of each clinical development process from the PHEIC declaration to the availability of therapeutics for COVID-19.

Figure 1 shows the definition of each clinical development process from the PHEIC declaration to the availability of therapeutics for COVID-19.

### 2.3 Analysis of clinical studies submitted for EUA application

The objectives, phases, and designs, including the endpoints of the studies submitted for regulatory review and the clinical data package of each EUA application, were confirmed based on CDER review documents (9–23) for each drug or biological therapeutic product. Additional information on the study design was obtained from clinical study registration sites (24, 25). Drugs and biological therapeutic products were classified according to whether they had already been approved or if clinical study data were available for other indications.

## 3 Results

### 3.1 Identification of therapeutic products for COVID-19

Table 1 shows the categories, administration routes, treatment targets, and dates of EUA submission/authorization of therapeutics for COVID-19 authorized in the US during the PHEIC declaration period. These included five anti-inflammatory products, three antiviral products, and six neutralizing antibodies. Among these, four anti-inflammatory products—baricitinib, propofol-lipuro 1% (B. Braun), tocilizumab, and anakinra—have been approved for other indications. Clinical studies of the remaining anti-inflammatory product, vilobelimab, and an antiviral product, remdesivir, are ongoing for other indications but were not approved when the pandemic was declared. Other agents have also been developed as novel molecular agents for treating COVID-19.

**Table 1:** Therapeutics for COVID-19 granted EUA in the US.

Category	Non-proprietary name	Brand name/ Company (example)	Administration route	Treatment target	Emergency use authorization		Note
					Submission date	Authorization date	
Anti-inflammatory	Baricitinib	Olumiant/Eli Lilly	Oral	Moderate to severe	2020/10/15	2020/11/19	JAK inhibitor is approved as a drug for rheumatoid arthritis, etc.
	Propofol-lipuro 1%	−/ B.Braun	IV	Moderate to severe	2021/2/1	2021/3/12	Approved as a drug for sedation in the EU.
	Tocilizumab	Actemra/ Roche	IV	Moderate to severe	2021/4/20	2021/6/24	Anti-IL-6 receptor antibody approved as a product for rheumatoid arthritis, etc.
	Anakinra	Kineret/Sobi	SC	Moderate to severe	2022/2/10	2022/11/8	IL-1 receptor antagonist approved as a drug for rheumatoid arthritis, etc.
	Vilobelimab	Gohibic/InflaRx GmbH	IV	Moderate to severe	2022/9/29	2023/3/29	IgG4 antibody binds to C5a. Clinical development for hidradenitis suppurativa etc., was progressing.
Anti-virus	Remdesivir	Veklury/Gilead	IV	Mild to severe	2020/4/16	2020/5/1	Clinical development for Ebola hemorrhagic fever was progressing.
	Molnupiravir	Lagevrio/MSD	oral	Mild to moderate	2021/10/8	2021/12/23	Newly developed product. Contraindicated for pregnant women, etc.
	Nirmatrelvir/ ritonavir	Paxlovid/Pfizer	oral	Mild to moderate	2021/10/21	2021/12/22	Newly developed product. Multiple contraindicated drugs.
Neutralizing antibody	Bamlanivimab	bamlanivimab/Eli Lilly	IV	Mild to moderate	2020/10/6	2020/11/9	Newly developed product.
	Casirivimab/ imdevimab	Ronapreve/Regeneron	IV	Mild to moderate	2020/10/8	2020/11/21	Newly developed product.
	Bamlanivimab/Etesevimab	Bamlanivimab-etesevimab/Eli Lilly	IV	Mild to moderate	2020/11/16	2021/2/9	Newly developed product.
	Sotrovimab	Xevudy/GSK	IV	Mild to moderate	2021/3/24	2021/5/26	Newly developed product.
	Tixagevimab/ cilgavimab	Evusheld/AstraZeneca	IM	Mild to moderate	2021/9/30	2021/12/8	Newly developed product.
	Bebtelovimab	bebtelovimab/Eli Lilly	IV	Mild to moderate	2022/1/7	2022/2/11	Newly developed product. It can be used by children over 12 years old.

### 3.2 Clinical development duration of therapeutic products for COVID-19

**Figure 2:**
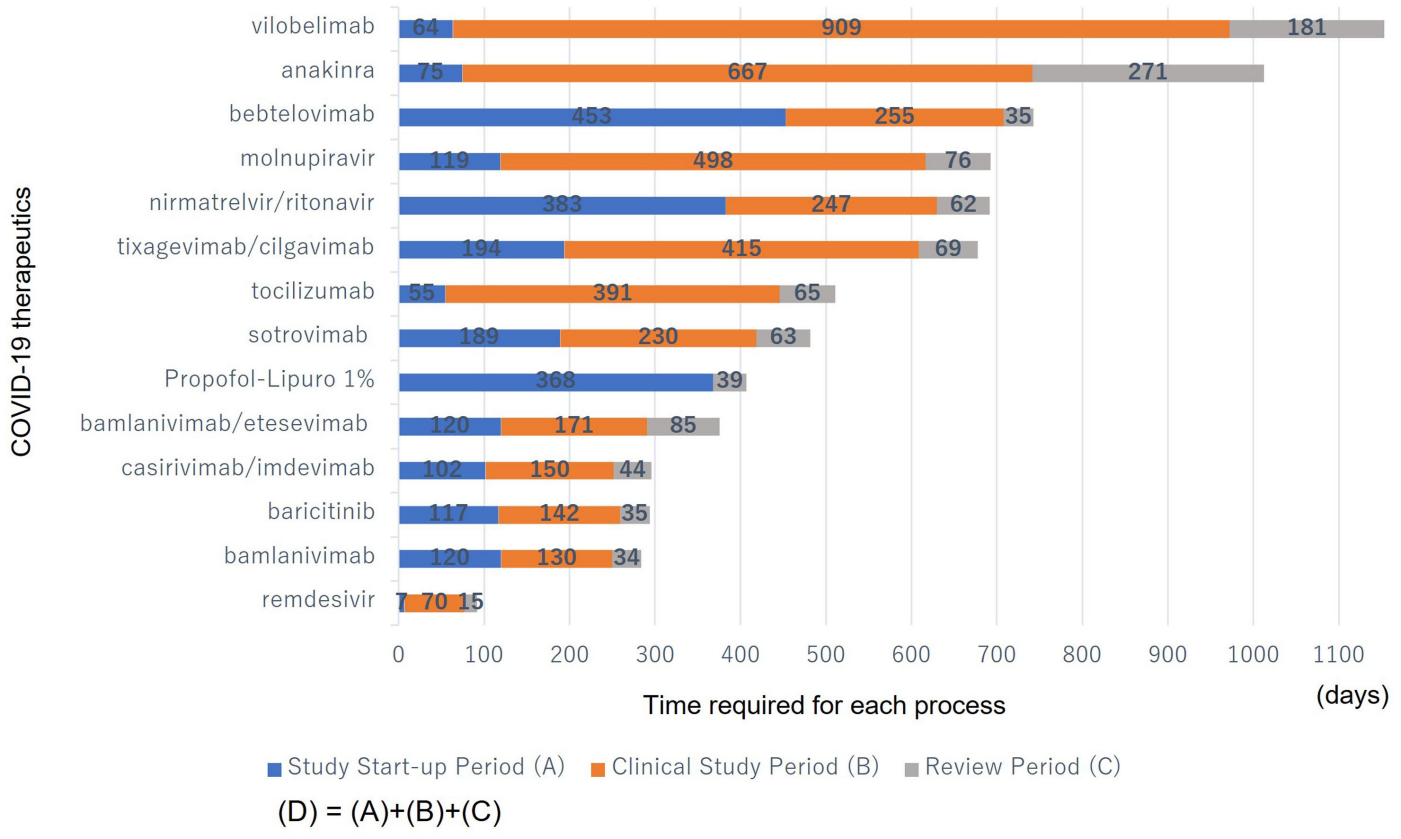
Time required for each process (A, B, and C) during the clinical development period (D) of the COVID-19 therapeutics. (A): From the date of the PHEIC declaration to the earliest clinical study registration date in the submitted studies. (B): From the earliest clinical study registration date in the submitted studies to the date of EUA submission. (C): From the date of EUA submission to the date of EUA issuance. They were sorted from bottom to top in descending order of the number of days until they became available.

Figure 2 shows the time required for each process (A, B, and C) during the clinical development period (D) of the COVID-19 therapeutics.

The study start-up period (A) ranged from 7 to 383 days; the clinical study period (B) from 0 to 909 days; the review period (C) from 15 to 271 days; and the total clinical development period (D), including (A), (B), and (C), from 92 to 1,154 days. Many products have a long clinical study period (B) as a proportion of the clinical development period (D).

### 3.3 Clinical studies included in the application data package of therapeutic products for COVID-19

#### 3.3.1 Overview

The number and phases of the clinical studies included in the application dossier for each product are shown in Table 2. For products already approved for other indications, the number of studies newly conducted for COVID-19 after the PHEIC declaration ranged from 0 to 4, with most being phase 3 studies. Previous clinical study data for other indications were not submitted. The number of studies on unapproved products that were under clinical development for other indications ranged from 1 to 9. The number of new studies conducted on COVID-19 after the PHEIC declaration ranged from 1 to 3, and most were phase 3 studies.

**Table 2:** Evaluation studies included in EUA applications.

COVID-19 therapeutics	Number of submitted studies^a^	Number of studies conducted for COVID-19^b^	Phase of submitted studies^c^		
Approved for other indications					
Baricitinib	1	1			Phase 3
Propofol-Lipuro 1%	0	0			
Tocilizumab	4	4			Phase 3
					Phase 3
				Phase 2/3^d^	
					Phase 3
Anakinra	2	2			Phase 3
					Phase 3
Median	1.5	1.5			
Mean	1.8	1.8			
SD	1.7	1.7			
Unapproved but under clinical development for other indications					
Remdesivir	9	3	Phase 1^e^		
			Phase 1^e^		
			Phase 1^e^		
			Phase 1^e^		
				Phase 2 for other indication^e^	
					Phase 3 for other indication^e^
					Phase 3
					Phase 3
					Phase 3
Vilobelimab	1	1		Phase 2/3	
Median	5.0	2.0			
Mean	5.0	2.0			
SD	5.7	1.4			
New molecular entity for COVID-19					
Bamlanivimab	7	7	Phase 1		
				Phase 2/3	
					Phase 3
					Phase 3
				Phase 2/3	
			Phase 1		
				Phase 2	
Casirivimab/ Imdevimab	6	6		Phase 2/3	
			Phase 1/2		
			Phase 1/2/3		
					Phase 3
			Phase 1		
			Unknown		
Bamlanivimab/ Etesevimab	12	12	Phase 1^f^		
				Phase 2/3^f^	
			Phase 1		
					Phase 3^f^
					Phase 3^f^
				Phase 2/3^f^	
			Phase 1^f^		
			Phase 1		
				Phase 2^f^	
				Phase 2	
			Phase 1		
			Unknown		
Sotrovimab	4	4			Phase 3
				Phase 2/3	
				Phase 2	
				Phase 2	
Tixagevimab/ Cilgavimab	3	3	Phase 1		
					Phase 3
					Phase 3
Nirmatrelvir/ Ritonavir	6	6	Phase 1		
			Phase 1		
				Phase 2/3	
			Phase 1		
			Phase 1		
			Phase 1		
Molnupiravir	6	6	Phase 1		
				Phase 2	
				Phase 2	
				Phase 2/3	
				Phase 2/3	
			Phase 1/2		
Bebtelovimab	1	1	Phase 1/2^f^		
Median	6.0	6.0			
Mean	5.6	5.6			
SD	3.2	3.2			
All products					
Median	4.0	3.5			
Mean	4.4	4.0			
SD	3.5	3.2			

Note: Submitted studies for each product were sorted in descending order based on the date of registration in the clinical study database.

a Number of studies submitted in the first EUA application.

b Number of studies initiated after the PHEIC declaration among submitted studies.

c Submitted studies and their phases.

d The same study was submitted for casirivimab/imdevimab.

e Implemented before the PHEIC declaration.

f The same study was submitted for bamlanivimab.

#### 3.3.2 New molecular entity

The number of submitted studies on new molecular entities varied by product (ranging from 1 to 12), with many of the application package including data from phase 1, 2, and 3 studies. The highest number of studies was submitted for bamlanivimab/etesevimab (*n* = 12); however, seven of them were also submitted for bamlanivimab alone. The smallest number of studies (*n* = 1) was submitted for bebtelovimab. Although a greater number of studies were submitted for investigational products being developed for other indications compared to approved products, no disparity emerged in the number of studies undertaken following the PHEIC declaration.

#### 3.3.3 Therapeutics with previous clinical trial data

For products that had already been approved for other indications, data from phase 1 and 2 studies for baricitinib, phase 1 studies for tocilizumab, and phase 1 and 2 studies for anakinra were not included in the application data, and no clinical study data were submitted for propofol-lipuro 1%. For products that were unapproved but under clinical development for other indications, Phase 1 data for vilobelimab were not included in the application. For products with new molecular entities, Phase 1 studies on sotrovimab, Phase 2 studies on tixagevimab/cilgavimab, and Phase 3 studies on bebtelovimab were excluded from the application data.

### 3.4 Clinical development plan for COVID-19 therapeutics

#### 3.4.1 Submitted studies

Table 3 presents data from the submitted studies for each COVID-19 therapy. Products approved for other indications were evaluated only based on the results of placebo-controlled comparative studies involving a large number of patients. Six of the eight products with new molecular entities, excluding sotrovimab and bebtelovimab, submitted results involving healthy volunteers, a small number of patients, and placebo-controlled studies. For sotrovimab, a phase 1 study in healthy volunteers was not conducted; however, its safety was confirmed in a placebo-controlled phase 2 study involving a small number of patients. Similarly, bebtelovimab was subjected to a placebo-controlled study involving a small number of patients, and its pharmacokinetics (PKs) were confirmed in patients but not in healthy volunteers. For some of the unapproved products under clinical development for other indications, results involving healthy volunteers, a small number of patients, and placebo-controlled studies were available, whereas others only submitted the results of studies involving a large number of patients.

**Table 3:** Data included in the submitted studies for each COVID-19 therapy.

COVID-19 therapeutics	Phase 1		Phase 2			Phase 3	
	Healthy Volunteer		Small number of patients		Large number of patients	Special group	
	Safety	PK	Dose finding	Safety & efficacy	Safety & efficacy compared to placebo	Safety & efficacy & PK	Drug interactions
Approved for other indications							
Baricitinib	–	–	–	–	Yes	–	–
Tocilizumab	–	–	–	–	Yes	–	–
Anakinra	–	–	–	–	Yes	–	–
Unapproved but under clinical development for other indications							
Remdesivir	Yes	Yes	–	Yes	Yes	–	–
Vilobelimab	–	–	–	–	Yes^a^	–	–
New molecular entity							
Bamlanivimab	Yes	Yes	–	Yes	Yes	Yes (children)	–
Casirivimab/imdevimab	Yes	Yes	Yes	Yes	Yes	–	–
Bamlanivimab/etesevimab	Yes	Yes	Yes	Yes	Yes	Yes (children, older adults)	–
Sotrovimab	–	–	–	Yes^b^	Yes	–	–
Tixagevimab/cilgavimab	Yes	Yes	–	–	Yes	Yes (older adults)	–
Nirmatrelvir/ritonavir	Yes	Yes	Yes	Yes	Yes	Yes (liver disorder, renal disorder)	Yes
Molnupiravir	Yes	Yes	Yes	Yes	Yes	Yes	–
Bebtelovimab	–^c^	–	–	Yes	Yes	–	–

a The study was conducted as a phase 2/3 study, but only the phase 3 part was evaluated, and the phase 2 part provided supporting data.

b For sotrovimab, there was no phase 1 study, and safety was confirmed in a study with a small number of patients.

c Although it was described as a Phase 1/2 study, it was a study on patients rather than healthy volunteers.

*Propofol was excluded from this table because no relevant studies were submitted.

This table is based on the CDER review documents at the time of the initial EUA and has not included additional data submitted since then.

#### 3.4.2 Characteristics of clinical development plan for COVID-19 therapeutics

**Figure 3:**
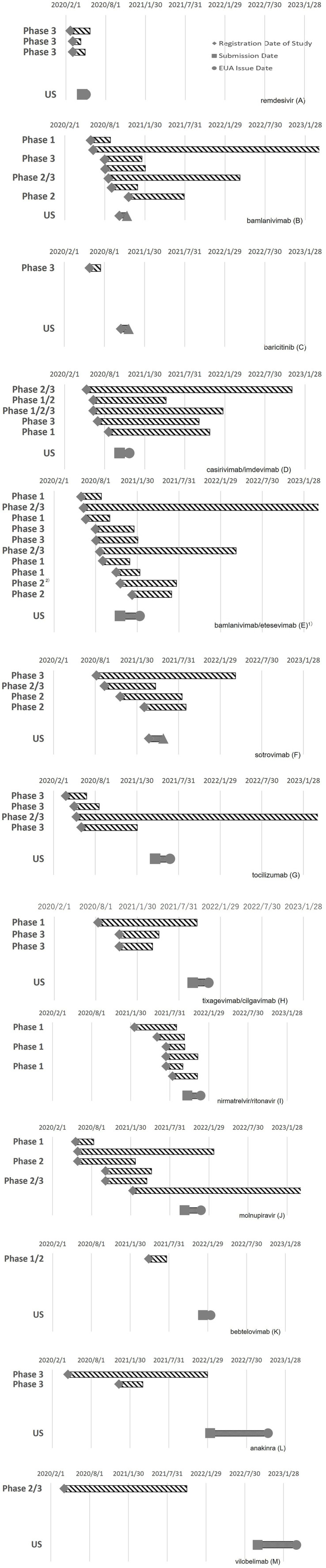
Timing of clinical studies and the EUA application/authorization of COVID-19 therapeutics: remdesivir **(A)**, bamlanivimab **(B)**, baricitinib **(C)**, casirivimab/imdevimab **(D)**, bamlanivimab/etesevimab **(E)**, sotrovimab **(F)**, tocilizumab **(G)**, tixagevimab/cilgavimab **(H)**, nirmatrelvir/ritonavir **(I)**, molnupiravir **(J)**, bebtelovimab **(K)**, anakinra **(L)**, and vilobelimab **(M)**.

No dose-finding studies have been performed for vilobelimab, bamlanivimab, sotrovimab, tixagevimab/cilgavimab, or bebtelovimab. Placebo-controlled comparative studies were conducted for all products, regardless of whether clinical study data for other indications were available before the pandemic. Data on specific populations, such as older adults, children, and pregnant women, were missing from the initial application data for many products. Many products, such as casirivimab/imdevimab (Figure 3D), bamlanivimab/etesevimab (Figure 3E), and sotrovimab (Figure 3F), have not been developed in the sequential phases of clinical studies. Many studies have been conducted with multiple objectives, such as phases 1/2 and 2/3, including those of bamlanivimab (Figure 3B), casirivimab/imdevimab (Figure 3D), nirmatrelvir/ritonavir (Figure 3I), and molnupiravir (Figure 3J). Large-scale clinical studies on remdesivir and baricitinib have been conducted by the government. An adaptive protocol design was used for studies on remdesivir and casirivimab/imdevimab. Several products, such as casirivimab/imdevimab and tocilizumab, use Selective Safety Data Collection (SSDC) to obtain safety information. In many cases, the EUA application was based on interim analysis data before the completion of the study and was reviewed on a priority basis.

### 3.5 Characteristics of the clinical development of each product

Figure 3 shows the timing of the clinical studies after the PHEIC declaration and EUA application/authorization of each product. The clinical development characteristics of each product obtained from the CDER review documents and clinical study registration sites are described below.

Remdesivir (Figure 3A): In addition to the clinical study data available before the PHEIC declaration, data from three studies conducted after the PHEIC declaration were submitted to the EUA. Three Phase 3 studies were launched almost simultaneously, one of which was a large-scale global study led by the US government. The EUA application was submitted approximately 2.5 months after the start of the studies. The adaptive protocol design and use of preliminary data may have contributed to its early application (2, 10, 26, 27).

Bamlanivimab (Figure 3B): Phase 1 and phase 2/3 studies were launched almost simultaneously, whereas phase 3 and phase 2/3 studies, including a government-led study, started approximately 2 months later. There was a phase 1 study that started later; this was a study to examine subcutaneous administration. Interim analysis data were used for the EUA application (11). Although seven studies were included in the clinical data package, clinical development was efficiently executed with a clinical study period (B) of 130 days, which was shorter than that for remdesivir.

Baricitinib (Figure 3C): Phase 3 study data were obtained after the PHEIC declaration. This Phase 3 study, led by the government, first confirmed the efficacy of remdesivir and then evaluated the efficacy and safety of baricitinib, which has anti-inflammatory effects. The application for EUA was submitted less than 5 months after the start of the clinical study for COVID-19 (12).

Casirivimab/imdevimab (Figure 3D): The earliest registration of clinical studies for the product occurred 102 days after the PHEIC declaration, which was earlier than that for any other product with a new molecular entity. Four studies, including phase 1/2/3 studies, were initiated at approximately the same time. The necessary human dose was estimated based on the results of non-clinical studies, and the treatment arms of casirivimab/imdevimab 1,200 mg and 4,000 mg each were established in the phase 1/2 part of the phase 1/2/3 study to evaluate the efficacy, safety, and PKs. As the interim analysis suggested that the target blood concentration would be achieved at 1,200 mg, enrollment in the 4,000 mg arm was discontinued, and the 600 mg arm was subsequently added. A phase 1 study was initiated later to examine subcutaneous administration. An adaptive protocol design and SSDC were adopted, and interim analysis data were used for EUA applications (13).

Bamlanivimab/etesevimab (Figure 3E): The number of clinical studies on bamlanivimab/etesevimab was 12, which was the highest among the target products. Phase 1 studies for bamlanivimab and etesevimab and phase 2/3 studies for the combination of bamlanivimab and etesevimab were initiated almost simultaneously. Approximately 1.5 months later, three phase 3 studies of the combination product were initiated, two of which were led by the government, and one was a study to investigate preventive effects. A phase 1 study was initiated to investigate the subcutaneous administration of bamlanivimab and etesevimab separately; a phase 2 study was used to confirm the efficacy of bamlanivimab and etesevimab in combination at various doses; a phase 2 study was started with bamlanivimab alone using real-world data as a control arm. Ten studies were initiated within approximately 6 months, but the clinical study period (B) was relatively short (171 days), and seven of the twelve studies were the same as those used for the application of bamlanivimab only. An interim data analysis was used in this application (14).

Sotrovimab (Figure 3F): Phases 3 and 2/3 studies were performed simultaneously. Although a phase 1 study was not conducted, safety was confirmed in the initial small number of patients in a phase 2/3 study. The dose was determined by estimating the required dose from non-clinical data and confirming that the target blood drug concentrations were achieved in a phase 3 study. PKs were confirmed in some patients in a phase 3 study. Interim analysis data were used for this application (15).

Tocilizumab (Figure 3G): Clinical studies of tocilizumab were second to start after the remdesivir study, and four clinical studies, including phases 3 and 2/3, were initiated at approximately the same time. Although the study start-up period (A) was short, the results of multiple comparative studies were inconsistent, and it took time to file the application for EUA (16).

Tixagevimab/cilgavimab (Figure 3H): The safety and PKs of the product in healthy volunteers were investigated in a phase 1 study, and two phase 3 studies were initiated approximately 3 months later. The doses for phase 3 studies were selected based on the results of non-clinical data. This product was approved for prophylactic use, and studies to confirm its treatment efficacy were conducted but were not included in the application (17).

Nirmatrelvir/ritonavir (Figure 3I): A phase 1 study was initiated first, and five studies, including phase 2/3, phase 1 studies in patients with renal or hepatic impairment, and phase 1 studies to investigate drug interactions, were initiated approximately 5 months later. This is a reliable way to proceed with the phase in order, but the application was filed approximately 3 months after the start of the phase 2/3 study while the study was underway. Although it took 383 days (A), the time required to launch the clinical study (period [B]) from the start of the clinical study to the application of EUA was relatively short (18).

Molnupiravir (Figure 3J): Three studies, including phases 1 and 2, were launched almost simultaneously. The dose–response was investigated in a phase 2 study and in phase 2 of another phase 2/3 study, and a dose of 800 mg and placebo were compared in a phase 3 study. Although the purpose of this study was not clearly identified, a phase 1/2 study was initiated for further investigation. Although the first clinical trial began quickly, it followed a gradual and steady developmental process that required substantial time (19).

Bebtelovimab (Figure 3K): A phase 1/2 study was submitted for EUA application. A monotherapy arm of bebtelovimab was added to the ongoing study of bamlanivimab and etesevimab. A phase 1 sub-study was included in this phase 1/2 study. It took time for the study start-up period (A) for this product because it was originally developed for the treatment of patients infected with SARS-CoV-2 variants, and the implementation period of the clinical study (B) was relatively short (20).

Anakinra (Figure 3L): Two phase 3 studies were conducted by a nonprofit organization, the Hellenic Institute for the Study of Sepsis for Anakinra. Although the first clinical trial was initiated rapidly, the second placebo-controlled study was initiated approximately 8 months later. The implementation period of the clinical study (B) and review period (C) were relatively long compared to those of the other products. This may be because the product was first applied for in the EU and then in the US, after approval by the EU (21).

Vilobelimab (Figure 3M): A phase 2/3 study was conducted to obtain supplemental data to support the available clinical study data for other indications of hidradenitis suppurativa and ulcerative pyoderma gangrenosum. Although the start-up period (A) was short, it took a reasonable period before the product became available for clinical use. This was probably due to the need to increase the number of patients after interim analysis and the time required to prepare for the application (22, 28).

## 4 Discussion

### 4.1 Lineup of therapeutic products

The products that received EUA for the treatment of COVID-19 included multiple categories of active ingredients, including antiviral, anti-inflammatory, and neutralizing antibodies; however, none of these were conclusive for the early resolution of the pandemic (29). The antiviral product remdesivir, which has been suggested as a potential therapeutic agent for various viral infections, first became available, followed by several neutralizing antibodies and anti-inflammatory products before the next antiviral product was released. However, the possible decreased susceptibility to various SARS-CoV-2 strains was soon pointed out for all neutralizing antibodies, and their use was then restricted (30, 31). The use of anti-inflammatory drugs is limited to patients with moderate-to-severe disease. Authorization for the next antiviral product, nirmatrelvir/ritonavir, was achieved approximately 600 days after remdesivir. The products with clinical study data for other indications were baricitinib, Propofol-lipro 1%, tocilizumab, and anakinra, which have been approved for other indications, and remdesivir and vilobelimab, which are investigational products being developed for other indications. All except remdesivir were anti-inflammatory agents.

This suggests that it might be difficult to control a pandemic at an early stage with a single product and that multiple therapeutic options are needed depending on the target population and stage of the pandemic. Individual products were first permitted within a limited population of patients; then, the scope was expanded, and the dose and administration of the products were sequentially improved. This implies that standard treatments are updated over a short period and that it would be beneficial to establish or verify new global- and regional-level frameworks to control and ensure the dissemination of information on standard treatments to enable emergency responses. Improving and refining such a framework would reduce the risks associated with the quick authorization of products before sufficient data are available.

### 4.2 Clinical data package for EUA applications

The application data packages for most products are assembled using conventional content. Placebo-controlled studies were included in the application dossiers for all products, regardless of whether clinical study data for other indications were available before the pandemic. A product with clinical study data for other indications would certainly be advantageous for early approval because substantial safety information on human participants is already available, and the process leading up to the initiation of clinical studies has already been completed. The only product authorized within 100 days of the PHEIC declaration, which was the goal of the 100 Days Mission, was remdesivir, for which clinical study data for other viral infectious diseases were available. Products with existing clinical study data can be readily subjected to efficacy studies; therefore, it is efficient, both in terms of time and resources, to promote large-scale clinical studies led by the government, where multiple promising candidate products are investigated simultaneously. To prepare for future pandemics, it is desirable to establish an international framework that can rapidly launch large-scale global clinical studies.

Placebo-controlled double-blind studies are needed, regardless of the clinical study data for other indications. Generally, placebo-controlled double-blind studies take more time to prepare than open-label studies because of additional processes, such as randomization and placebo formulation manufacturing. As the preparation period may be shortened by having an unblinded pharmacist at the clinical site instead of preparing the placebo formulation, information on whether unblinded staff can be assigned should be included. To initiate clinical studies promptly, organizing candidate agents for various epidemics during regular intervals is essential. To this end, it is desirable to include approved drugs as well as investigational products in each region. Depending on the degree of shortage in each category, there should be a mechanism through which funds are provided to meet the needs of various types of agents.

### 4.3 Analysis of each clinical development process

Regardless of clinical study data availability for other indications, clinical studies must be conducted on many products to obtain the data necessary for marketing authorization, including EUA. Here, we discuss the characteristics of each clinical development process and explore areas for improvement.

#### 4.3.1 Study start-up period (A)

Products with new molecular entities include neutralizing antibodies, such as bamlanivimab/etesevimab, and antivirals, such as molnupiravir and nirmatrelvir/ritonavir. It is presumed that neutralizing antibodies were newly developed against target molecules in SARS-CoV-2, and antiviral compounds were selected from the molecular library accessible to each pharmaceutical company; however, there was no relationship between the mechanism of action and the length of the study start-up period (A). Typically, candidates are narrowed down step-by-step to select the final compound. However, for COVID-19 therapeutic development, multiple steps are assumed to proceed simultaneously, accepting investment risks. One potential way to shorten this process could be to deregulate Investigational New Drug (IND) applications. It would also be beneficial if regulators organize and disclose the regulatory flexibilities they have applied to the development of individual medical products for COVID-19 during the pandemic and reflect them in guidance documents on product development for future pandemics, including possible deregulation of IND applications, such as minimum requirements for quality and non-clinical data according to the risk/benefit ratio of the pandemic.

The selection of study sites is one of the most time-consuming steps in clinical study preparation. Although conducting the study in a limited region could shorten the period, it may be necessary to expand the study region due to factors such as faster patient enrollment, the inclusion of a broader range of viral variants, and the potential need for region-specific study data for authorization. It would be beneficial if a list of study sites that can participate in the clinical studies of infectious diseases in each country/region were available beforehand so that many regions could participate without delaying the overall study timeline. The feasibility survey and participation request could be efficiently carried out.

#### 4.3.2 Clinical study period (B)

Although most clinical data packages of products for COVID-19 with new molecular entities were assembled in the same manner as those for normal clinical development, the phases did not proceed sequentially, and multiple objectives were included in one study, such as phase 1/2 and phase 2/3 studies. For example, four studies on casirivimab/imdevimab were initiated almost simultaneously: phase 2/3, phase 1/2, phase 1/2/3, and phase 3 studies. Furthermore, phase 3 and phase 2/3 studies on sotrovimab have been initiated almost simultaneously. In standard development, a clinical study is designed based on the results obtained in the previous phase of the study/studies to ensure patient safety and avoid futile investment owing to suboptimal choices. During the pandemic, the disadvantages of missed opportunities seemed more serious, and the speed of development was prioritized over steadiness. The pharmaceutical company planned early investments from potential non-clinical and early phase results while ensuring minimal safety, which was accepted by the regulatory authority.

Dose selection varies according to the product category. Dose-finding studies were performed for all antiviral products but not for neutralizing antibodies, which is one of the reasons for the difference in the length of the clinical development period. This may be due to differences in the strictness of dose selection between small-molecule products and antibody products and the difficulty of PK prediction because the antiviral products molnupiravir and nirmatrelvir/ritonavir are both oral drugs. Since the clinical study period (B) comprises a large portion of the clinical development period (D), it can be said that neutralizing antibodies and injections are advantageous for the early approval of products with a new molecular entity. Regarding the speed of clinical development, neutralizing antibodies are the next most advantageous after products with clinical study data for other indications; however, there is a risk that they may quickly lose their effectiveness against viruses that are prone to mutations, as observed during the COVID-19 pandemic. This experience may discourage sponsors from developing neutralizing antibodies during the next pandemic, but neutralizing antibodies are still needed because they are expected to be highly effective, and new antiviral products take time to develop. Thus, incentives may be necessary for the development of products that are expected to be effective but may have a short lifespan.

Adaptive protocol designs, such as remdesivir and casirivimab/imdevimab, have been applied in clinical studies. During the COVID-19 pandemic, it was necessary to determine the timing of evaluation and the target population for clinical studies before the characteristics of SARS-CoV-2 infection, such as natural course and high-risk population, were clarified. Therefore, it was necessary to collect appropriate data for evaluation as a clinical study proceeded and to make full use of the results owing to the high urgency level. Interim analyses have also been used in several product studies to design the protocol for the next study or to submit data for EUA. Several products, such as casirivimab/imdevimab and tocilizumab, employ SSDC to collect safety information that contributes to streamlining clinical trial operations. The SSDC approach eliminates the collection of non-serious adverse event information for products with an established safety profile for which additional safety information from clinical studies is not expected to affect the safety profile (32).

Therefore, improvements in efficiency, such as the utilization of non-clinical study data to determine the clinical dose, use of an adaptive design, use of interim analyses for early phase transition decisions or EUA application, and selective monitoring, are possible options depending on urgency and risk. Assessing the impact of the COVID-19 pandemic and refining the application conditions for each measure will help prepare for future pandemics. This will help sponsors make early investment decisions while reducing the time spent on emergency consultations with regulatory authorities.

#### 4.3.3 Review period (C)

All EUA applications during the pandemic were evaluated over a shorter period than usual. The length of the review period was not affected by the availability of clinical study data for other indications. It was inferred that there was close communication between applicants and regulatory authorities in the early stages. This approach was somewhat effective, but an unusual response to the pandemic. Improvements in the efficiency of procedures for the EUA, communication between applicants and regulators, and harmonization of application data among regions are expected for future pandemics.

### 4.4 Proposals for the future pandemics

Based on the clinical development of therapeutics during the COVID-19 pandemic, we can offer the following recommendations.

As the process leading up to the initiation of clinical studies has already been completed and safety information is available, products with clinical study data for other indications have the advantage of obtaining early approval. Therefore, a platform for sharing and organizing information on candidate products, including those under investigation, in each region should be established during normal times. At the same time, strategies for the early approval of new molecular entities are crucial, as products with clinical study data for other indications may not be sufficiently effective against new infectious diseases. Approving a product promptly, even if initially limited to a specific population, can help control the epidemic more effectively. The range of treatment targets can be gradually expanded as more data becomes available through clinical studies and real-world use. To achieve this goal, it is necessary to create a cooperative framework for managing and disseminating information at various levels among stakeholders, such as international organizations, global regulatory authorities, and academic societies. Furthermore, a global collaboration framework must be established to enable the swift launch of large-scale international joint clinical studies in each region or country. To encourage pharmaceutical industries to make early decisions on starting and progressing clinical development of therapeutic products for a pandemic, regulatory authorities—ideally through globally harmonized guidelines—should demonstrate regulatory flexibility based on the risks and benefits of the pandemic and the conditions for applying advanced clinical trial methods, such as adaptive design.

## 5 Limitations

This study focused on the clinical development of therapeutic products; thus, it did not address issues such as manufacturing and distribution, which are key elements affecting the time to clinical availability. All products discussed in this study were targeted for acute-phase treatment of COVID-19; however, long-term administration may need to be considered depending on the target disease, and the timeline will likely differ in this case.

## 6 Conclusion

This study revealed the characteristics of the clinical development of therapeutic products for COVID-19. Several products of different categories became available over a short period, and clinical data packages for EUA were generally constructed in the same manner as for normal clinical development. However, each phase was not conducted in sequence because of the prioritizing speed of clinical development, and various advanced clinical development methods have been adopted. Based on these results, we recommend establishing a global collaboration framework for organizing information on candidate products during normal times and sharing information on changes in dosage and target populations of each product as development progresses during the pandemic. We also recommend the establishment of a global platform to launch large-scale global clinical trials quickly. Regulatory authorities are expected to clarify the conditions for applying regulatory flexibility, which will encourage the pharmaceutical industry to make early decisions regarding clinical development.

## Data Availability

The original contributions presented in the study are included in the article/supplementary material, further inquiries can be directed to the corresponding author.

## References

[1] World Health Organization (WHO). COVID-19 public health emergency of international concern (PHEIC), global research and innovation forum: Towards a research road map. Available online at: https://www.who.int/publications/m/item/covid-19-public-health-emergency-of-international-concern-(pheic)-global-research-and-innovation-forum (Accessed August 4, 2024).

[2] Saint-Raymond A Sato J Kishioka Y Teixeira T Hasslboeck C Kweder SL. Remdesivir emergency approval: a comparison between the U.S., Japanese, and EU systems. Expert Rev Clin Pharmacol. (2020) 13:1095–101. doi: 10.1080/17512433.2020.1821650, PMID: 32909843

[3] Brown DG Wobst HJ Kapoor A Kenna LA Southall N. Clinical development times for innovative drugs. Nat Rev Drug Discov. (2022) 21:793–4. doi: 10.1038/d41573-021-00190-9, PMID: 34759309 PMC9869766

[4] US Food and Drug Administration. Emergency use authorization. About Emergency Use Authorization (EUAs) Available online at: https://www.fda.gov/emergency-preparedness-and-response/mcm-legal-regulatory-and-policy-framework/emergency-use-authorization (Accessed August 4, 2024).

[5] US Food and Drug Administration. Emergency use authorization of medical products and related authorities; guidance for industry and other stakeholders; availability (2017). Available online at: https://www.federalregister.gov/documents/2017/01/13/2017-00721/emergency-use-authorization-of-medical-products-and-related-authorities-guidance-for-industry-and (Accessed August 4, 2024).

[6] United Kingdom Government. 100 days mission to respond to future pandemic threats (2021). Available online at: https://www.gov.uk/government/publications/100-days-mission-to-respond-to-future-pandemic-threats (Accessed August 4, 2024).

[7] US Food and Drug Administration. Emergency use authorizations for drugs and non-vaccine biological products. Available online at: https://www.fda.gov/drugs/emergency-preparedness-drugs/emergency-use-authorizations-drugs-and-non-vaccine-biological-products (Accessed August 4, 2024).

[8] World Health Organization. WHO director-general's opening remarks at the media briefing (2023). Available online at: https://www.who.int/news-room/speeches/item/who-director-general-s-opening-remarks-at-the-media-briefing---5-may-2023 (Accessed August 4, 2024).

[9] US Food and Drug Administration. CDER scientific review documents supporting emergency use authorization for drug and biological therapeutic products/COVID-19. Available online at: https://www.fda.gov/drugs/coronavirus-covid-19-drugs/cder-scientific-review-documents-supporting-emergency-use-authorizations-drug-and-biological (Accessed August 4, 2024).

[10] US Food and Drug Administration. Emergency use authorization (EUA) for remdesivir, an unapproved product Center for Drug Evaluation and Research (CDER) review. Available online at: https://www.fda.gov/media/155772/download?attachment (Accessed August 4, 2024).

[11] US Food and Drug Administration. Emergency use authorization (EUA) for bamlanivimab 700 mg IV Center for Drug Evaluation and Research (CDER) review. Available online at: https://www.fda.gov/media/144118/download?attachment (Accessed August 4, 2024).

[12] US Food and Drug Administration. Emergency use authorization (EUA) for baricitinib for the unapproved use of an approved product Center for Drug Evaluation and Research (CDER) review. Available online at: https://www.fda.gov/media/144473/download?attachment (Accessed August 4, 2024).

[13] US Food and Drug Administration. Emergency use authorization (EUA) for casirivimab and imdevimab Center for Drug Evaluation and Research (CDER) review. Available online at: https://www.fda.gov/media/144468/download?attachment (Accessed August 4, 2024).

[14] US Food and Drug Administration. Emergency use authorization (EUA) for Bamlanivimab 700 mg and Etesevimab 1400 mg administered together Center for Drug Evaluation and Research (CDER) review. Available online at: https://www.fda.gov/media/146255/download?attachment (Accessed August 4, 2024).

[15] US Food and Drug Administration. Emergency use authorization (EUA) for Sotrovimab 500 mg Center for Drug Evaluation and Research (CDER) review. Available online at: https://www.fda.gov/media/150130/download?attachment (Accessed August 4, 2024).

[16] US Food and Drug Administration. Emergency use authorization (EUA) for tocilizumab for the unapproved use of an approved product Center for Drug Evaluation and Research (CDER) review. Available online at: https://www.fda.gov/media/150748/download?attachment (Accessed August 4, 2024).

[17] US Food and Drug Administration. Emergency use authorization (EUA) for EVUSHELD (Tixagevimab 150 mg and Cilgavimab 150 mg injection co-packaged for intramuscular use) Center for Drug Evaluation and Research (CDER) review. Available online at: https://www.fda.gov/media/155107/download?attachment (Accessed August 4, 2024).

[18] US Food and Drug Administration. Emergency use authorization (EUA) for Paxlovid (nirmatrelvir tablets co-packaged with ritonavir tablets) Center for Drug Evaluation and Research (CDER) review. Available online at: https://www.fda.gov/media/155194/download?attachment (Accessed August 4, 2024).

[19] US Food and Drug Administration. Emergency use authorizations (EUA) for molnupiravir 200 mg capsules Center for Drug Evaluation and Research (CDER) review. Available online at: https://www.fda.gov/media/155241/download?attachment (Accessed August 4, 2024).

[20] US Food and Drug Administration. Emergency use authorizations (EUA) for bebtelovimab (LY-CoV1404) Center for Drug Evaluation and Research (CDER) review. Available online at: https://www.fda.gov/media/156396/download?attachment (Accessed August 4, 2024).

[21] US Food and Drug Administration. Emergency use authorization (EUA) for Kineret (anakinra) for unapproved use of an approved product Center for Drug Evaluation and Research (CDER). Available online at: https://www.fda.gov/media/163546/download?attachment (Accessed August 4, 2024).

[22] US Food and Drug Administration. Emergency use authorizations (EUA) for vilobelimab (IFX-1) Center for Drug Evaluation and Research (CDER) review. Available online at: https://www.fda.gov/media/167044/download?attachment (Accessed August 4, 2024).

[23] US Food and Drug Administration. Emergency use authorizations (EUA) for propofol-Lipuro 1% injectable emulsion for infusion Center for Drug Evaluation and Research (CDER) review. Available online at: https://www.fda.gov/media/147466/download?attachment (Accessed August 4, 2024).

[24] National Library of Medicine ClinicalTrials.gov. About. Available online at: https://clinicaltrials.gov/about-site/about-ctg (Accessed August 4, 2024).

[25] EudraCT. European Union Drug Regulating Authorities Clinical Trials Database. Available online at: https://eudract.ema.europa.eu/ (Accessed August 4, 2024).

[26] Ohmagari T. Adaptive COVID-19 clinical trials. Yokohama, Japan. Paper presented at the 43rd Annual Meeting of the Japanese Society of Clinical Pharmacology and Therapeutics; (2022).

[27] Beigel JH Tomashek KM Dodd LE Mehta AK Zingman BS Kalil AC. Remdesivir for the treatment of Covid-19 – final [report]. N Engl J Med. (2020) 383:1813–26. doi: 10.1056/NEJMoa2007764, PMID: 32445440 PMC7262788

[28] Vlaar APJ Witzenrath M van Paassen P Heunks LMA Mourvillier B de Bruin S. Anti-C5a antibody (vilobelimab) therapy for critically ill, invasively mechanically ventilated patients with COVID-19 (PANAMO): a multicentre, double-blind, randomised, placebo-controlled, phase 3 trial. Lancet Respir Med. (2022) 10:1137–46. doi: 10.1016/S2213-2600(22)00297-1, PMID: 36087611 PMC9451499

[29] Aleem A Samad ABA Vaqar S. Emerging variants of SARS-Cov-2 and novel therapeutics against coronavirus (COVID-19). Available online at: https://www.ncbi.nlm.nih.gov/books/NBK570580/ (Accessed August 4, 2024). 34033342

[30] Cavazzoni P. US Food and Drug Administration (2024). Coronavirus (COVID-19) update: FDA limits use of certain monoclonal antibodies to treat COVID-19 due to the omicron variant. Available online at: https://www.fda.gov/emergency-preparedness-and-response/counterterrorism-and-emerging-threats/coronavirus-disease-2019-covid-19 (Accessed August 4, 2024).

[31] The National Institutes of Health. COVID-19 treatment guidelines. Anti-SARS-CoV-2 monoclonal antibodies. Available online at: https://www.covid19treatmentguidelines.nih.gov/therapies/antivirals-including-antibody-products/anti-sars-cov-2-monoclonal-antibodies/ (Accessed August 4, 2024).

[32] US Food and Drug Administration. Determining the extent of safety data collection needed in late-stage premarket and postapproval clinical investigations (2016). Available online at: https://www.fda.gov/files/drugs/published/Determining-the-Extent-of-Safety-Data-Collection-Needed-in-Late-Stage-Premarket-and-Postapproval-Clinical-Investigations.pdf (Accessed August 4, 2024).

